# Endoplasmic reticulum—Phagosome contact sites from the cradle to the grave

**DOI:** 10.3389/fcell.2022.1074443

**Published:** 2022-12-22

**Authors:** Mahlegha Ghavami, Gregory D. Fairn

**Affiliations:** ^1^ Department of Pathology, Dalhousie University, Halifax, NS, Canada; ^2^ Department of Biochemistry and Molecular Biology, Dalhousie University, Halifax, NS, Canada

**Keywords:** phagocytosis, phagosome, calcium, cholesterol, ER contact sites, ORP1L, STIM-ORAI, phagolysosome

## Abstract

Phagocytosis is a key component of the innate immune system used to ingest apoptotic cells and microorganisms for their destruction and recycling of macromolecules and the presentation of antigens to adaptive immune system cells. The newly formed vacuole or nascent phagosome undergoes a maturation process reminiscent of the classical endocytic maturation process, reaching a highly degradative phagolysosome stage before its tubulovesicular breakdown into lysosomes. The process is highly regulated and can be disrupted by various pathogenic organisms. The exchange of proteins, lipids, and other metabolites between organelles, including maturing phagosomes, is enabled by two processes, vesicular and non-vesicular transport at membrane contact sites (MCS). For decades the specific role(s) of the endoplasmic reticulum (ER) in phagocytosis has been the subject of much debate. In parallel, the last two decades have seen a burst in research on the numerous roles of ER contact sites and resident proteins in all aspects of organelle biology. Here, in this minireview, we describe ER-phagosome contact sites’ functions from the early stages of particle engulfment to the phagolysosome dissolution into lysosomes. We also discuss several aspects of ER–phagosome contact sites that remain to be explored.

## Introduction

Phagocytosis is a specialized form of endocytosis used to engulf particulate matter such as invading pathogens or apoptotic bodies > 0.5 μm in diameter (Gordon, 2016). Professional phagocytes, including macrophages, dendritic cells, and neutrophils, use phagocytosis to eliminate microorganisms and to present antigens to cells of the adaptive immune system (Gray and Botelho, 2017; Henson, 2017). In addition to its role in the innate immune response, phagocytosis contributes to homeostasis and tissue remodeling by eliminating apoptotic bodies. Thus, phagocytosis is critical to support tissue homeostasis and innate and adaptive immunity (Gray and Botelho, 2017; Lim et al., 2017).

Phagosomes display heterogeneity in size as the dimensions of the nascent vacuole are dictated by the particle being internalized (Keller et al., 2017). Phagosomes are “on-demand” organelles absent from resting cells but quickly form due to receptor-mediated internalization of prey and later disappear once the target has been degraded (Goodridge and Underhill, 2017; Uribe-Querol and Rosales, 2020). Conceptually, the life cycle of the phagosome consists of three stages: i) phagocytosis, particle engulfment, and the formation of a nascent phagosome, ii) maturation, the conversion to the highly degradative phagolysosome, and iii) phagolysosome “resolution,” a series of tubulovesicular events leading to the dissolution of the phagolysosome and the reformation of lysosomes (Levin-Konigsberg et al., 2019).

**FIGURE 1:**
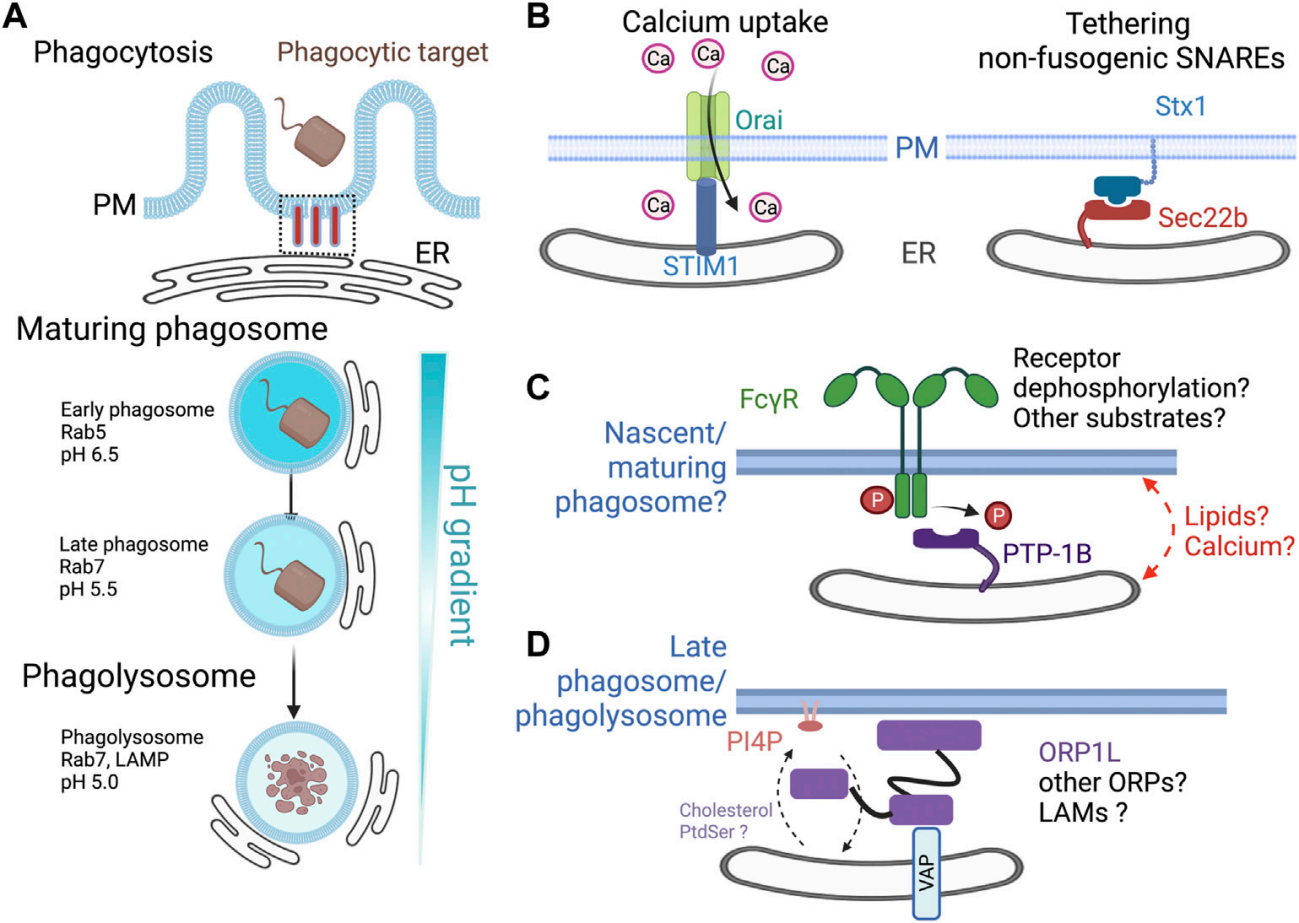
Described and potential roles for ER-phagosome contact sites. **(A)** Phagosome formation and maturation. The ER is in close apposition to the base of the phagocytic cup during the initial particle engulfment through the breakdown of the mature phagolysosome. Like the endocytic pathway, phagosomes undergo a maturation process from early to late, reaching a phagolysosome stage. These stages are distinguished by Rab5 or Rab7 and a decrease in luminal pH. **(B)** During phagosome formation, the ER forms contact sites with the base of the phagocytic cup. STIM1-ORAI interact in trans, allowing for the influx of extracellular calcium. Sec22b, a non-fusogenic SNARE localizes to the sites of phagocytosis whose over-expression impairs particle uptake. **(C)** PTP-1B is a tail-anchored ER protein that can dephosphorylate growth factors receptor and other proteins at ER contact sites. Since numerous proteins, including the FcγRs, undergo tyrosine phosphorylation during phagocytosis, we speculate that PTP-1B may function at an ER-phagosome contact site. **(D)** Late phagosomes/phagolysosomes resynthesize PI4P on the cytosolic leaflet of the membrane. The Rab7 effector ORP1L interacts with the ER proteins of the VAP-A and VAP-B and serves as both a tether and a lipid transfer protein. ORP1L transfers PI4P from the phagolysosome membrane to the ER membrane, where the PI4P phosphatase, SAC1, dephosphorylates it. ER, endoplasmic reticulum; STIM1, stromal interaction molecule 1; ORAI, calcium release-activated calcium modulator 1; PM, plasma membrane; PTP-1B, protein tyrosine phosphatase 1B; ORP1L, oxysterol-binding protein-related protein 1L; VAP, VAMP-associated protein; PI4P, phosphatidylinositol 4-phosphate.

Phagocytosis is initiated by activating cell surface opsonic or scavenger receptors upon interaction with ligands on target particles. The activation of receptors and the extension of pseudopods result in the formation of the phagocytic cup, which originates from the plasma membrane (PM) but whose growth is supported by the focal exocytosis of endosomes and lysosomes. Advancing pseudopods wrap around the prey, forming a nascent phagosome (Greenberg and Grinstein, 2002). Newly formed phagosomes undergo acute remodeling by an orderly sequence of fusion with organelles of the endocytic and biosynthetic pathways while maintaining a nearly constant surface area by concurrent fission of membrane vesicles (Pitt et al., 1992; Desjardins et al., 1994; De Chastellier et al., 1995). This results in the extensive reorganization and maturation of the membrane and contents of the phagosome (Figure 1A), which is characterized by obtaining lysosomal enzymes, reactive oxygen intermediates production, and luminal acidification (Tjelle et al., 2000). The highly degradative phagolysosome can hydrolyze complex molecules from the ingested prey (proteins, complex carbohydrates, and lipids) into smaller building blocks (amino acids, simple sugars, and lipids) for recycling and reuse. In contrast, indigestible materials remain trapped within the phagolysosome or can undergo exocytosis to release the particles into the extracellular environment (Gray and Botelho, 2017). Once the contents within the phagolysosome have been degraded, a variety of poorly understood tubulovesicular processes facilitate the dissolution of the vacuole to regenerate lysosomes that support subsequent phagocytosis and elimination of prey.

Studies from the last two decades have demonstrated the importance of MCS between two, and now even three, organelles (Levine, 2004; Levine and Loewen, 2006; Boutry and Kim, 2021). In general, MCS represent regions where organelles come into proximity, typically within 10 nm–30 nm, but do not directly fuse (Helle et al., 2013; Valm et al., 2017). MCS are relatively stable structures maintained by tethering proteins or pairs of proteins that work together to link the two membranes (Filippin et al., 2003). Functionally, MCS support the bidirectional movement of molecules, especially lipids and calcium, and control the access of enzymes for their substrates including PTP-1B and potentially the Sac1 phosphatidylinositol 4-phosphate phosphatase (Henne et al., 2015; Henne, 2016; Scorrano et al., 2019; Prinz et al., 2020), although whether Sac1 works in trans or cis remains unresolved (Zewe et al., 2018; Venditti et al., 2019). Numerous reviews are available on the general features of MCS, and we refer readers to Scorrano et al. (2019) which includes additional details on what defines a MCS. As lipid remodeling, calcium release, and phosphotyrosine signaling all support phagosome formation and maturation, it is possible that ER-phagosome contact sites may have many important functions in the life cycle of the phagosome.

### Is “ER-mediated phagocytosis” simply an ER–phagocytic cup membrane contact site?

The internalization of large phagocytic targets, such as apoptotic cells and fungal organisms, or numerous smaller targets, requires the delivery of internal membranes to the site of phagocytosis (Uribe-Querol and Rosales, 2020). Proteomic studies using latex beads and microorganisms demonstrated that isolated phagosomes contain a variety of ER-resident proteins (Gagnon et al., 2002). Furthermore, this study and a variety of others using electron microscopy could demonstrate that the ER comes into close contact with the PM (Guermonprez et al., 2003; Houde et al., 2003). However, another group revealed that despite the close apposition of the ER to the site of phagocytosis, the two organelles were not directly fusing (Touret et al., 2005) and that endosomes and lysosomes were the primary sources of the “extra” membrane required to support robust phagocytosis. However, given the ever-growing literature on ER contact sites, the earlier observations and proteomic data raise the question of whether ER contact sites are formed with a portion of the PM actively engaged in phagocytosis and if the MCS is important for the process.

### ER-phagocytic cup contact sites

Using J774 murine macrophage cells early studies supported the ER–PM fusion model of phagocytosis showed that the ER-localized SNARE proteins, Syntaxin18, and Sec22b promote phagocytosis (Becker et al., 2005; Hatsuzawa et al., 2006). However, no evidence has been provided to demonstrate that the ER can directly fuse with the PM in this or other cellular contexts (Saheki and De Camilli, 2017). One possibility is that Sec22b interacts with Syntaxin 1 or another SNARE, and together these two proteins form an ER-PM tether (Figure 1B). However, it has also been demonstrated that over-expression of Sec22b can act as an inhibitor of phagocytosis, implying that excess or mislocalized non-fusogenic SNARE interactions/tethering can block particle uptake (Hatsuzawa et al., 2009). Many questions remain regarding the role of these non-fusogenic SNARE interactions. Evidence from neurons demonstrates that Sec22b contributes to the expansion of the PM (Petkovic et al., 2014), a process that could conceivably assist with particle engulfment. This study revealed that the yeast Sec22 interacts with yeast oxysterol-binding proteins Osh2 and Osh3 to support lipid transfer and thus the membrane expansion. By analogy, it is conceivable that mammalian Sec22b may facilitate the localized exchange of lipids. In support of this concept, a recent preprint has been released examining the role of the mammalian oxysterol binding protein-related protein 8 (ORP8) and Sec22b in mouse embryonic fibroblasts made phagocytic through the expression of Fcγ2A receptor (Santos et al., 2022). This study demonstrates that Sec22b supports ER-phagosome contact sites, influences the metabolism of phosphoinositides, and phagosome maturation in this non-professional phagocyte. Together, while the exact roles of Sec22b remain unknown, the growing body of literature suggests that it functions as part of an MCS tether.

### Calcium influx and STIM-Orai1 activation during particle uptake

Both global and local calcium signals influence phagosome formation and maturation, including actin cytoskeleton remodeling and phagolysosome fusion (Nunes and Demaurex, 2010). Phospholipase C activation at the sites of phagocytosis results in the local hydrolysis of phosphatidylinositol 4, 5-*bis*phosphate (PI4,5P_2_), producing diacylglycerol and inositol *tris*phosphate (IP_3_). The IP_3_ can then bind its receptor in the ER, causing a release of Ca^2+^ from its lumen. Upon depletion of the ER Ca^2+^ store, the ER-resident protein stromal interaction molecular 1 (STIM1) undergoes a series of conformational changes, allowing it to interact with plasmalemmal resident calcium release-activated calcium channel protein 1 (termed ORAI) (Wu et al., 2006). Light and electron microscopy, coupled with sophisticated genetic and molecular biology techniques, demonstrate that STIM1-mediated ER contacts promote the Ca^2+^ transients recorded during phagosome formation and early maturation (Nunes et al., 2012) (Figure 1B). In addition, the results suggest that binding of STIM1 to ORAI channels facilitates actin filament depolymerization from the base of the phagocytic cup (Garin et al., 2001; Buschow et al., 2012; Campbell-Valois et al., 2012). Importantly, these findings demonstrate that MCS form at the base of the phagocytic cup during the initial stages of phagocytosis. Since STIM1 and ORAI interact in *trans* at ER-PM MCS, their interaction may have a secondary role of stabilizing contact sites which may allow other proteins to function.

### PTP-1B and MCS—A role in the life cycle of the phagosome?

Phosphotyrosine signals drive most, if not all, forms of phagocytosis. There are numerous excellent reviews on the signal transduction pathways at play during phagocytosis, so we will only highlight a few general features. The best-studied phagocytosis pathway involves Fcγ Receptors, which bind to the opsonin immunoglobulin G on particles. Clustering of Fcγ Receptors (FcγR) activates Src family kinases (SFKs) which phosphorylate tyrosine residues in the cytosolic region of the FcγR. The phosphorylated tyrosine residues then serve as a docking site for Syk tyrosine kinase, which further phosphorylates itself and additional adaptors and transducers of the signal. The full complement of tyrosine phosphatases involved in turning off phagocytic signaling is not currently known.

PTP-1B is bound to the ER *via* a C-terminal transmembrane anchor (Frangioni et al., 1992). PTP-1B is best known for its ability to dephosphorylate SFKs, epidermal growth factor receptor (EGFR), and insulin receptor (IR) (Bakke and Haj, 2015). Using substrate-trapping mutants and fluorescence energy transfer, researchers have demonstrated that PTP-1B functions at ER–endosome contact sites and potentially ER–PM contact sites (Anderie et al., 2007; Eden et al., 2010; Yue et al., 2019). As the ER-phagosome contact site resembles the ER-endosome contact site, the biological significance of PTP-1B could be to downregulate FcγR signaling or other phagocytic receptors. In this regard, failure to efficiently dephosphorylate the phospho-ITAMs of the FcγR could potentially lead to prolonged signaling from the nascent or early phagosomes. Additionally, and by analogy to the EGFR, PTP-1B may impact the relative abundance of FcγRs since their targeted degradation in multivesicular bodies requires timely dephosphorylation. Whether this would enhance or hinder the rates of IgG-mediated phagocytosis in macrophages is unclear. Continued signaling by Src family kinases and Syk would result in the phosphorylation and activation of a variety of effectors and scaffolding proteins (Lowell, 2011), which may be limiting if the proper spatial and temporal coordination is lost. Syk kinase is also a substrate of PTP-1B, but whether this occurs at an ER-phagosome contact site has not been deduced (Figure 1C). Given our knowledge of MCS, PTP-1B, and phagocytosis, it is reasonable to hypothesize that PTP-1B may be dephosphorylating FcγR, SFKs, Syk, or other substrates at an ER–phagosome contact site.

Phagocytic receptors are not the only putative PTP-1B substrates in the phagosome. Través and colleagues previously demonstrated that murine or human macrophage deficient in PTP-1B have greater responses to the polyinosinic:polycytidylic acid (a TLR3 ligand) and lipopolysaccharide (a TLR4 ligand) (Través et al., 2014). This study used only the purified forms of these ligands. However, it is conceivable that TLR4 and other receptors could be locally activated at the site of phagocytosis and that PTP-1B may downregulate this signal during or following internalization. Clearly, the role of PTP-1B in phagosome formation and maturation is worth investigating and may reveal unappreciated aspects of signal transduction in macrophages and potentially other phagocytic cells.

### Non-vesicular transfer of lipids at MCS

In eukaryotic cells, most lipids are synthesized in the ER, with a few others produced in the mitochondria or Golgi apparatus. However, many lipids reside primarily in other compartments, especially the PM. The cell has two ways to move lipids between organelles; vesicular and non-vesicular transport. For the sake of brevity, we will focus on the non-vesicular transport pathways. Most lipids are too hydrophobic to spontaneously leave one membrane bilayer, transit an aqueous environment, and insert into another bilayer. Even the comparatively short distances at MCS are insufficient for the free diffusion of lipids to be biologically relevant. Instead, eukaryotes have evolved a variety of cytosolic proteins, which can move lipids one-by-one between organelles and others that act mostly as channels or troughs to allow for faster, less selective transfer (Jansen et al., 2011; Toulmay and Prinz, 2011; Egea, 2021). Many of these proteins function at ER-PM contact sites or ER-endosome/lysosome contact sites. Unfortunately, very little work on these proteins exists in the context of the life cycle of the phagosome. However, we anticipate that many proteins will likely have a role in the process.

### ORP1L and ER-phagolysosome contact sites influence many aspects of the phagolysosome

One lipid transfer protein studied in the context of phagosome maturation is oxysterol binding protein (OSBP) related protein 1 L (ORP1L). ORP1L is unique among the OSBP/ORP family as it contains an N-terminal ankyrin domain that allows it to bind to active Rab7 found on late endosomes, lysosomes, and the phagosome equivalents (Johansson et al., 2005). Like many ORPs, ORP1L also contains an FFAT-motif, which allows it to bind to the ER-resident proteins VAMP-associated protein (VAP) A and B (Loewen et al., 2003; Loewen and Levine, 2005; Rocha et al., 2009) By binding active Rab7 and VAPs, ORP1L mediates ER-lysosome contact sites. One role of ORP1L is to act as a cholesterol sensor and regulator of lysosomal transport (Van Der Kant et al., 2013; Wijdeven et al., 2016; Zhao and Ridgway, 2017). When cholesterol levels in late endosomes/lysosomes are low, ORP1L is in an open conformation, allowing contact with Rab7 and VAP (Van Der Kant et al., 2013). However, when cholesterol accumulates in the endosomal membrane, ORP1L dissociates from ER and disrupts the MCS. During maturation, the lysosomes and phagosomes move centripetally toward the microtubule organizing center (MTOC) (Harrison et al., 2003; Eng et al., 2007). This dynein-mediated process is required for lysosome fusion. Rab7 connects the phagosome to microtubules *via* its effectors, Rab7-interacting lysosomal protein (RILP) and ORP1L (Harrison et al., 2003; Rocha et al., 2009). RILP recruits the dynein-dynactin complex with the aid of ORP1L to promote minus-end transport along microtubules. This centripetal migration of phagosomes is essential for the phagolysosome maturation (Jordens et al., 2001; Van Der Kant et al., 2013). Additionally, the Rab7-RILP-dynein-dynactin complex facilitated the movement of these compartments to support the maturation process of phagosomes since it brings them into proximity to lysosomes (Harrison et al., 2003; Wijdeven et al., 2016). To what extent does the phagosome movement toward the MTOC require ORP1L? Still, based on the analogy to other organelles, including autophagosomes and lysosomes, we suspect that it is a minimum playing a supportive role. However, it is abundantly clear that active Rab7 and ORP1L are associated with the phagosome at this stage.

ORP1L has been shown to mediate the transport of cholesterol and PI4P at various ER-contact sites with late endosomes, lysosomes, and phagolysosomes. For instance, in non-phagocytic cells, in the absence of low-density lipoprotein (LDL), ORP1L mediates cholesterol transport from the ER, where it is synthesized, to late endosomes (Eden et al., 2016). Alternatively, when cells are supplied with LDL cholesterol, ORP1L can facilitate its transfer in the opposite direction (Zhao et al., 2020). In macrophages, ORP1L localizes at the MCSs between the ER-phagolysosome contact sites, where it mediates the transport of PI4P from phagolysosomes to the ER (Levin-Konigsberg et al., 2019) (Figure 1D). Live imaging revealed that the disappearance of PI4P from phagolysosomes was impaired in ORP1L-deficient macrophages. This study could not find clear evidence of cholesterol counter-transfer. However, it has been reported that cholesterol tends to increase with phagosome maturation (Rai et al., 2016). In this study, the authors noted that late phagosomes have more cholesterol than early phagosomes and that cholesterol facilitated the clustering of flotillin, Rab7, and dynein (Rai et al., 2016). The authors suggest that clustering of dynein may facilitate interactions with microtubules and thus promote movement towards the MTOC. Currently, the relative importance of ORP1L as a cholesterol sensor and regulator of dynein activity and the ability of cholesterol transfer proteins to generate cholesterol-rich nanodomains that also facilitate dynein-mediated transport are unclear. This and other possible functions of cholesterol in the late phagosome and phagolysosome warrant further investigations.

**FIGURE 2:**
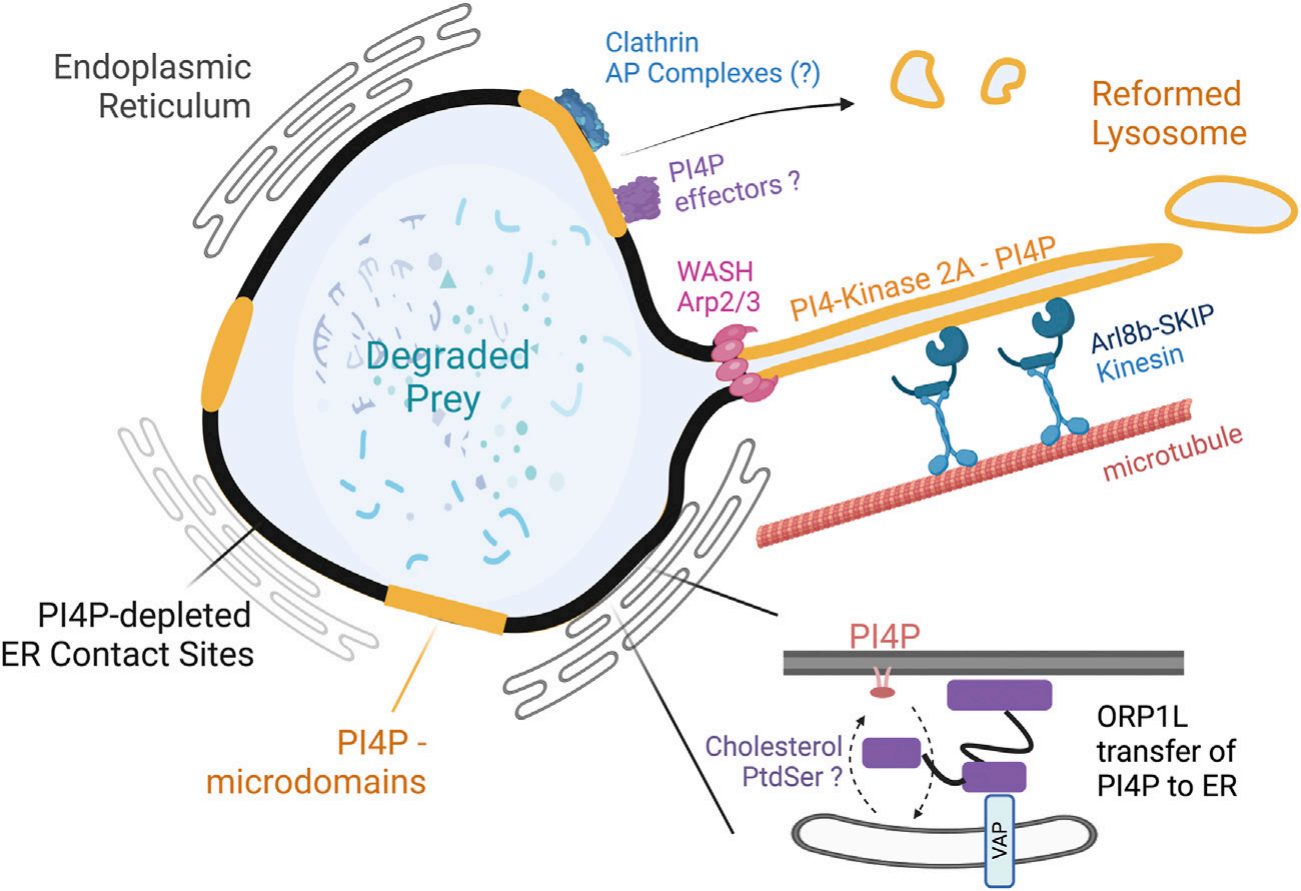
PI4P domains and ORP1L-mediated contact sites support the tubulovesicular breakdown of phagolysosomes. The phagolysosome acquires PI4P through the acquisition of PI4-Kinase 2A. ORP1L at contact sites transfers PI4P to the ER, where it is dephosphorylated by Sac1. Regions of the phagolysosome not associated with ORP1L or the ER maintain PI4P. The PI4P domains aids in the recruitment of SKIP, Arl8b, and kinesin motors to produce long tubules emanating from the phagolysosome. The Arp2/3 complex and WASH aid in the scission of tubules. The process reduces the size of the phagolysosome and reforms lysosomes. A second clathrin-dependent pathway is proposed to be involved in the dissolution of bacteria containing phagosomes. Whether the clathrin-dependent pathway requires PI4P, ER-contact sites, or an adaptor complex is currently unclear.

The examination of PI4P and ORP1L in the phagolysosome revealed striking heterogeneity; regions containing ORP1L and in contact with the ER were devoid of PI4P, whereas the areas free of ORP1L and the ER were rich in PI4P (Figure 2). Live cell imaging using lattice light sheet and electron microscopy revealed robust and long tubules emanating from the phagolysosome once the prey had been degraded. The PI4P positive domains on the phagolysosome serve as “hotspots” to recruit SKIP/PLEKHM2, further recruiting Arl8b and kinesin motors that collectively draw tubules away from the phagolysosome. Importantly, in cells devoid of ORP1L, the PI4P remained continuous on the phagolysosomes, which impaired tubule formation and phagolysosome breakdown (Levin-Konigsberg et al., 2019). Inhibiting the formation of ORP1L-mediated contacts also inhibited phagolysosome resorption, suggesting a role for the MCS in the conclusion of phagocytosis (Figure 2). The findings demonstrate that ORP1L-VAP contacts promote resolution in two distinct ways: the generation of PI4P-rich and depleted microdomains spatially enriches tubulation machinery; and by providing a physical tether that immobilizes the phagolysosome to mechanically support tubulation and fission (Zhao and Ridgway, 2017; Levin-Konigsberg and Grinstein, 2020). In addition to repopulating lysosomes consumed during phagosome maturation, the tubules can carry processed antigens and fuse with MHC Class II compartments for subsequent antigen presentation (Mantegazza et al., 2013). Consequently, it is plausible that phagolysosomal membrane resolution is coupled to antigen presentation mechanisms, especially in dendritic cells.

## Discussion

Despite being a fundamental process involved in innate immunity, our understanding of the role of MCSs in the life cycle of the phagosome is in its infancy. Given that most, if not all, phagocytic pathways rely on phosphotyrosine signaling, deciphering the role of PTP-1B in regulating the process seems like a fruitful area of investigation. Additionally, considering the extensive membrane remodeling of the limiting membrane of the maturing phagosome, it would be useful to determine which lipid transfer proteins are involved in these processes. Further, since many pathogenic organisms, such as mycobacterium, can alter phagosome maturation and establish a replicative niche, it is tempting to speculate that MCSs may be hijacked to support the growth of microorganisms.

Do ER MCSs mediate phagolysosome repair? A variety of phagocytic prey can cause physical damage to the phagosome, including toxin-expressing bacteria, uric acid crystals, and protein fibrils. Recently, an elaborate lysosomal membrane repair pathway was described in non-phagocytic cells. This pathway requires phosphatidylinositol 4-Kinase type 2 alpha (PI4K2a) to generate PI4P and recruit ORP protein family members OSBP, ORP1L, ORP9, ORP10, and ORP11 (Radulovic et al., 2022; Tan and Finkel, 2022). These proteins transfer PI4P to the ER as the driving force of moving cholesterol or phosphatidylserine, respectively. Subsequently, the ORP-mediated transfer of lipids, in particular phosphatidylserine, leads to the recruitment of autophagy-related 2 (Atg2), another type of lipid transfer protein, which aids in the repair of the lysosomal membrane (Tan and Finkel, 2022). The role of this response in repairing phagosomes or phagolysosomes has yet to be determined.

Do other pathways involved in phagolysosome breakdown require PI4P and MCS? Although very little is known about the dissolution of phagosomes compared to earlier phagocytic stages, it has a critical reliance on ER contact site-mediated lipid transfer. Once phagolysosomes have fulfilled their microbicidal and degradative duties, they must be removed to facilitate future rounds of phagocytosis (Lancaster et al., 2021). For each phagosome formed, a significant proportion of the plasma membrane, endosomes, and lysosomes are consumed along with any integral and lumen enzymes. Thus, the timely breakdown of this now-exorbitant organelle has the benefit of regenerating smaller organelles. However, is the requirement for ORP1L and ER contact sites dependent on the size of the phagolysosome? For instance, Lancaster et al. demonstrated that small bacteria-containing phagolysosomes undergo fragmentation *via* clathrin-dependent vesicle budding and fission. Importantly, this pathway resulted in the regeneration of lysosomes that supported the degradative capacity of future phagolysosomes. This makes teleological sense as the alternative would require the *de novo* synthesis and trafficking of hydrolytic enzymes and the vacuolar ATPase responsible for the acidification of the endosomes.
